# Modifications in the Free Gingival Graft Technique: A Systematic Review

**DOI:** 10.7759/cureus.58932

**Published:** 2024-04-24

**Authors:** Bader Fatani, Hissah Alshalawi, Ahmed Fatani, Raghad Almuqrin, Mohamed S Aburaisi, Fatin Awartani

**Affiliations:** 1 Dentistry, King Saud University, Riyadh, SAU; 2 Dentistry, Ministry of Health, Riyadh, SAU; 3 Statistics, Alrawad Institution, Riyadh, SAU; 4 Periodontics and Community Dentistry, King Saud University, Riyadh, SAU

**Keywords:** outcome, approach, technique, modified, modification, free gingival graft

## Abstract

Free gingival grafts (FGGs) have developed beyond covering exposed roots to improve the width and thickness of gingival tissue. While traditional FGGs have shown a high success rate and are easy to apply, they have some drawbacks, such as potential aesthetic concerns and bulky appearance. Recent advancements in FGGs have been explored, with different modifications proposed to overcome these limitations, including partly epithelialized FGGs (PE-FGG), gingival unit grafts, and epithelialized subepithelial connective tissue grafts. This systematic review aims to evaluate published case reports that discuss the utilization of modified approaches to FGG treatment and their outcome.

## Introduction and background

Gingival recession, where the gingival margin moves downwards from the tooth's surface, can lead to aesthetic concerns, increased tooth sensitivity, root caries risk, and increased blood flow in the pulp. While the exact cause of gingival recession is not fully understood, factors like oral hygiene standards suggest that mechanical and anatomic elements may be involved. Various surgical techniques are available to address this issue, such as free grafts like free gingival grafts (FGGs) and subepithelial connective tissue grafts, along with pedicled grafts like lateral and coronal grafts. Free gingival grafts, originating from Bjorn in 1963 and later termed FGG by Nabers in 1966, have advanced from solely covering exposed roots to enhancing gingival tissue width and thickness. Despite their high success rate and straightforward application, traditional FGGs have limitations, including potential aesthetic issues and bulky appearance [1]. Recent advancements in FGG have been under review, with various modifications suggested to address FGG's limitations. These adjustments encompass partly epithelialized FGG (PE-FGG), gingival unit graft, and epithelialized subepithelial connective tissue graft [2]. Multiple previous case reports discussed the use of modified approaches in free gingival graft [1-6]. However, there is still no systematic review summarizing the modifications used. Thus, this systematic review aims to review published case reports discussing the use of modified techniques in free gingival graft treatment.

## Review

Elaboration

**Figure 1:**
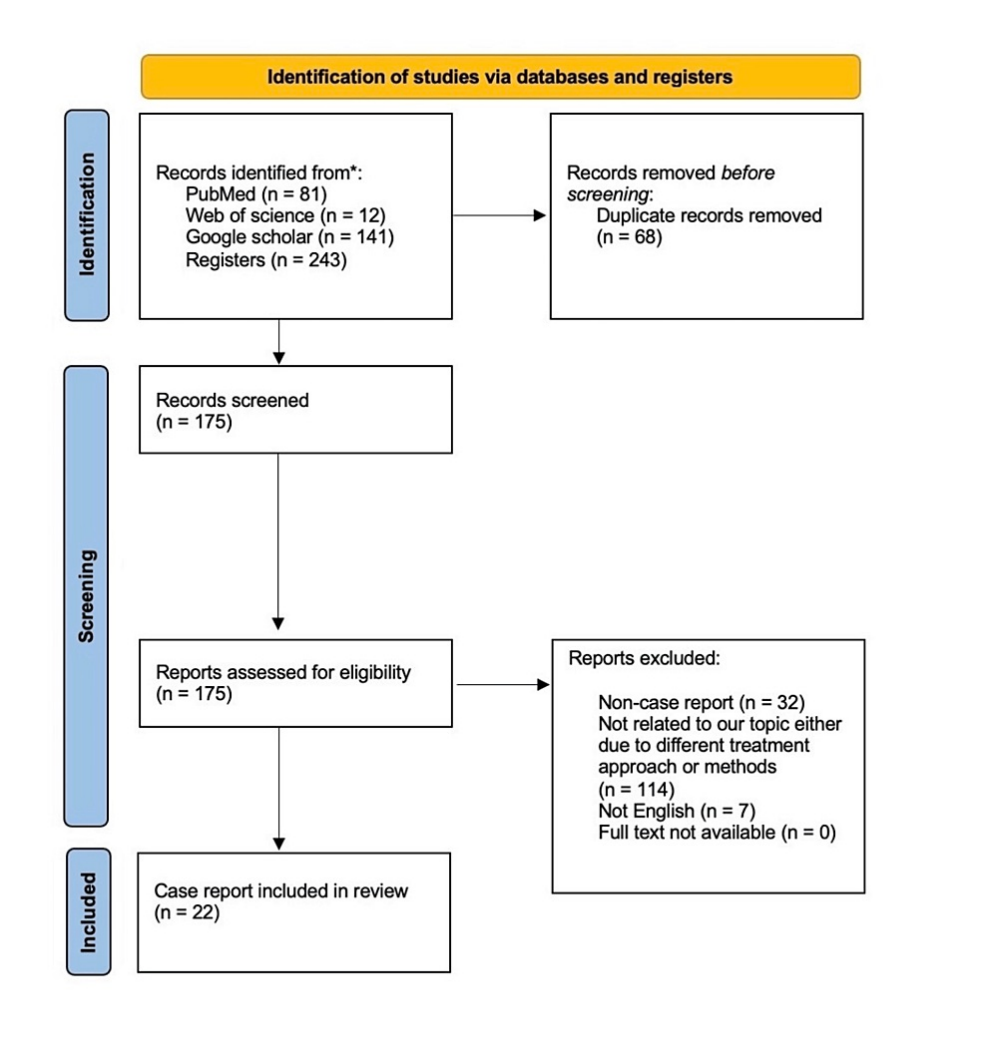
Flow chart of included studies. * The following databases were included as they are the main source of case reports in the literature, other databases were excluded due to insufficient case reports available.

This systematic review followed the guidelines of the Preferred Reporting Items for Systematic Review and Meta-Analyses protocols (PRISMA 2020). The acronym PICOS will be used to answer this systematic review question, "What are the different modifications used in free gingival grafts and their outcomes?" PICOS stands for P = Patients requiring periodontal graft rehabilitation, I = Modified approach of free gingival graft, C = Control group if applicable, and O = Improved periodontal measurements such as keratinized tissue, gingival growth, esthetic, etc. The search strategy was applied to PubMed, Google Scholar, and Web of Science without time restrictions. Figure 1 illustrates the flow chart for this study.

Eligibility criteria and selection process

For the selection process of the articles, we included in this systematic review all reports that discussed a modification approach for FGGs and were defined as inclusion criteria. The exclusion criteria were as follows: (1) Non-case reports, (2) Out-of-scope reports, (3) Did not discuss FGGs, (4) Non-English-language articles, and (5) Articles with no full text available. This systematic review did not include other systematic reviews, clinical trials, prospective studies, lab studies, review articles, or retrospective studies. The articles were selected in two stages. In the first step, the reviewers evaluated the titles and abstracts of the papers found after applying the search strategy according to the eligibility criteria to select the articles to read fully. In the second step, the reviewers independently assessed the articles selected for full reading according to the eligibility criteria. We performed an extensive online search and literature review to assess the various modifications in FGGs. Google Scholar, PubMed, and Web of Science databases were searched for articles published up to April 2024. Free gingival grafts, modifications, and outcomes were the terms included in the search. There was no specific timeframe for the included papers, and the selected articles were screened using the PRISMA 2020 statement and flow diagram. Electronic files were organized using the Mendeley reference management tool.

Data extraction

Mendeley served as the system for managing references and collecting all papers from the literature search. Reviewers assessed studies based on PRISMA 2020 guidelines. Duplicates were eliminated. Abstracts were screened for suitability by at least two reviewers. Full-text articles subsequently underwent a thorough review by four reviewers focusing on research designs and results. Cited studies were also reviewed, with disputes resolved through discussion. Articles were included only if all reviewers agreed they met all criteria. Data from selected articles were gathered and organized in an Excel spreadsheet according to the following criteria: (a) Author, (b) Year of publication, (c) Participant, (d) Technique used, and (e) Results or outcome.

Results 

Most case reports were published after the year of 2015. The papers reflected cases that indicated the use of an FGG. The case reports included in this systematic review were from global populations from different countries. All reported cases focused on the modified techniques used in FGGs. Among these, five papers discussed more than one case. The case reports discussed the patient, the modified technique used, and the outcome of the approach used in FGG procedures. In most case reports, the subjects were females. The case reports included both healthy and unhealthy subjects. According to most case reports, modifying the FGG procedure can enhance the outcome of periodontal treatment. These outcomes varied depending on which modified technique was used and included successful root coverage, enhanced gingival color matching, more keratinized tissue, the promotion of new attachments, and greater healing of the donor area. Table 1 summarizes the discussed case reports.

**Table 1:** Summary of discussed case reports. A-PRF: advanced platelet-rich fibrin

Authors	Year of publication	Participant/s	Technique used	Outcomes
De et al. [1]	2023	Two cases	After placing implants, significant bone loss with a shallow gingival pocket and minimal attached gingival tissue were treated in two individuals using a combination of an apically positioned flap and an expanded mesh-free gingival graft.	All treatment sites healed well with better gingival pocket depth, tissue thickness, and width of tissue around the implants. Four months post-grafting, a pleasing aesthetic result was achieved.
Carcuac et al. [2]	2021	Three cases	A modification of the FGG technique is discussed. The goal of the modification is to enhance blood flow from the treated area to the graft, ultimately improving the procedure’s predictability and results.	Complete or near-complete coverage of roots was accomplished in difficult cases of gingival recession.
Singh et al. [3]	2023	35-year-old female	A 1.5-mm-thick graft was taken from the hard palate on the left side and tailored to fit the recipient site size determined by a template. The graft was meticulously placed on the prepared bed and aligned toward the bone, leaving 3.5 mm of exposed bone. Thinner sutures secured the graft following the Holbrook and Oschenbein techniques.	An increase of 3 mm in the height of the keratinized gingival tissue is achieved.
Danskin et al. [4]	2023	30-year-old male	Mucogingival surgery using a tunneling technique. The goal was to maintain the position of the flap without pushing it forward, keeping the interdental papillae intact to improve blood flow. An FGG was taken, processed, then passed through the tunnel and secured.	Using a de-epithelialized connective tissue graft through tunneling while preserving the deeper muscle attachment is a conservative approach to enhance the appearance of lingual recession defects.
Shah et al. [5]	2015	Three cases	In the first procedure, a thin split-thickness graft from the palate was placed between the interdental tissues. In the second case, the graft from the palate extended from the first premolar to the first molar. The remaining recession area was covered with the connective tissue segment of the PE-FGG obtained using a modified double incision technique. In the third case, the graft was taken from the palate, guided by the recession size, following a variation of the single incision method by Hürzeler and Weng, keeping a small piece of the gingiva epithelium intact.	In all three cases, successful root coverage and improved color matching compared to FGGs were achieved.
Mayta et al. [6]	2021	71-year-old male	Positioning the FGG, holes were created in it using a 5-mm trephine-type milling cutter. The graft was placed in the affected implants and secured with a basic technique using Glicosorb polyglycolic acid. A palatal plate was crafted to shield the donor area.	Three months later, the mucosa looked healthy and keratinized, particularly around implants previously identified with mucositis, leading to satisfactory outcomes for the patient.
Lee et al. [7]	2018	36-year-old female patient	Using a 3–12 MHz transducer designed for intraoral use on a US imaging device, the area from the greater palatine foramen was examined. Blood flow in the greater palatine artery was easily found through Doppler imaging.	The Doppler mode showed blood flow, and color coding distinguished arteries and veins. Pulsating flow enabled accurate artery observations in real-time.
Park et al. [8]	2023	33-year-old male smoker	A submerged method used partially de-epithelialized FGGs to change the appearance of soft tissues in the anterior part of the maxilla.	The technique, involving partially de-epithelialized FGGs underwater, showed it could widen keratinized tissue and cover roots effectively on multiple teeth, even with thin palatal mucosa.
Alrmali et al. [9]	2023	Two cases	A new method utilized an FGG shaped similar to an inverted T to partially cover roots.	Achieving the partial coverage of roots, enhancing the compromised area between teeth, and increasing the width of keratinized tissue in RT3 defects.
Cholan et al. [10]	2014	32-year-old female	An FGG taken to widen the attached gingiva used electrocautery techniques. The evaluation included patient-reported discomfort at the donor site and a clinical increase in keratinized and attached gingival width.	Electrosurgery for harvesting FGGs has clear benefits and can be advocated as a practical substitute for conventional scalpel methods.
El Nahass et al. [11]	2015	37-year-old female	Due to the visible metal display, more soft tissue was added around the implant using an FGG from the palate. The graft had two de-epithelialized extensions for blood supply and color matching. It was then placed with pouches for extensions.	Treatment results displayed superior outcomes in both the amount and quality of new soft tissue.
Kinaia et al. [12]	2019	21-year-old female	The patient had 5 mm of gingival recession on a tooth that had been previously rotated. A partially de-epithelialized FGG was used to increase keratinized gingiva and decrease root exposure.	Partially de-epithelialized FGGs can be utilized to enhance keratinized gingiva in cases of lingual recession while providing sufficient coverage for the root.
Harris et al. [13]	1998	45-year-old female	The vestibular modification procedure described in this study differs from gingivoplasty or excisional methods used to remove a prior FGG. It serves as the opposite of vestibuloplasty, aiming to remove keratinized tissue below the desired mucogingival junction and restore the natural vestibular shape at a higher level on the tooth.	The combination of surgical techniques and prosthetics led to an exceptional outcome including growth of papilla on both sides of the defect following initial surgery.
Srinivas et al. [14]	2015	29-year-old male	A combination of an FGG and the commercially accessible fibrin–fibronectin sealing system (Tissucol(®)) is utilized to treat Miller's Class II gingival recession.	A fibrin–fibronectin sealing system (Tissucol®) offers an extra tool in periodontal treatment and can be effectively applied in periodontal surgeries to promote new attachment and faster postoperative recovery.
Imano et al. [15]	2019	38-year-old female	A modified technique with an FGG is used to increase both the vertical and horizontal amount of keratinized gingiva before placing a dental implant.	The dental implant was placed, with a 3-mm increase in vertical keratinized gingiva.
Kulkarni et al. [16]	2014	18 patients	Platelet-rich fibrin was prepared, compressed, and applied to a palatal wound with a periodontal pack in one group, while the other group only received a periodontal pack.	Using platelet-rich fibrin as a dressing is a successful way to boost the healing of the palatal donor area, leading to lower post-operative complications.
Falabella et al. [17]	2018	Two cases	Each patient received an FGG with a coronally positioned flap during treatment.	Using the FGG effectively augmented keratinized tissue and satisfactorily covered the recession.
Ibraheem [18]	2018	59-year-old female	An FGG was inserted at the location to expand the keratinized tissue area, followed by the application of platelet-rich fibrin to enhance the healing process. Cyanoacrylate adhesive secured the platelet-rich fibrin.	The utilization of platelet-rich fibrin speeds up the recovery of FGGs. Enhanced tissue manipulation can be accomplished by expanding keratinized tissue during surgical interventions.
Henriques et al. [19]	2011	Case of severe Miller’s Class II gingival recession	The patient underwent a two-stage surgery combining an FGG and connective tissue grafting. Initially, an FGG was conducted to achieve sufficient keratinized tissue. Three months later, a connective tissue graft (CTG) achieved root coverage.	Findings showed that FGG increases keratinized tissue and CTG provides root coverage with reduced recession after 16 months.
Vijayendra et al. [20]	2011	30-year-old male	Following scaling, the initial procedure to widen the attached gingiva was done with an FGG. Two months later, connective tissue from the palate was harvested and positioned to address the remaining defect.	Two months after the operation, there was a 3-mm increase in the width of the attached gingiva and 35% root coverage. By 6 months post-operation, there was a notable rise in the width of the attached gingiva, CAL, with decreased recession size.
Rath et al. [21]	2022	22-year-old female	The patient had a Miller's Class II recession, with a high frenulum attachment and a shallow mouth opening. The condition was addressed with an FGG, and the donor area was covered with advanced A-PRF.	Using an A-PRF membrane as a palatal dressing seems to hasten healing at the donor site.
Vieira et al. [22]	2017	36-year-old female	Two methods of FGG involving the use of oral screws for graft attachment were used. Traditional FGG was performed on teeth 44 to 46, while partly epithelialized FGG was performed on teeth 34 to 36 on the other side.	Partially epithelialized FGGs were noted to offer superior cosmetic outcomes, with the use of stabilizing screws proving to be a straightforward and efficient method.

Discussion 

Recent improvements in free gingival graft (FGG) have been undergoing evaluation, with different changes proposed to overcome the limitations of using FGG [2]. Chetana et al. conducted a study to compare the effectiveness of gingival unit graft (GUG)/ gingival unit transfer (GUT) and free gingival graft in treating gingival recession. They found that the GUG technique provided a higher percentage of sites with complete root coverage compared to the free gingival graft method [23]. Moreover, Deo et al. conducted a study to assess the effectiveness of free gingival grafts in treating localized gingival recessions of Miller Class I and II. Their findings suggested that while free gingival grafts can yield significant results, the outcome is greatly influenced by the selection of cases and the expertise of the operator. In situations where there is insufficient width of the attached gingiva and depth of the vestibular fornix, free gingival grafts may be considered a favorable alternative [24]. Meza and colleagues conducted a study to explore whether using platelet-rich fibrin (PRF) after harvesting palatal-free gingival grafts could enhance healing, reduce pain, and better control postoperative bleeding in the palatal region. They suggested that the application of PRF membrane in the palatal area following free gingival graft harvesting might be advantageous [25]. According to Silva et al. study which sought to find evidence-based methods to enhance healing or safeguard the harvested palate in patients receiving gingival grafts. They found several promising interventions such as cyanoacrylate adhesive, platelet-rich fibrin (PRF), and a combination of palatal stents with healing agents that could potentially improve patient outcomes [26]. A study by Gusman et al. evaluated the healing process and pain levels after using PRF in palatal wounds post-free gingival graft (FGG) removal. Their findings suggested that using PRF in palatal donor sites of FGG could potentially reduce postoperative pain and accelerate complete wound healing [27]. Gul and colleagues conducted a longitudinal clinical study to investigate the relationship between creeping attachment and baseline recession depth following the placement of free gingival grafts (FGG) below class I, II, and III Miller's recession defects. The study revealed that positioning free gingival grafts below recession areas led to a notable reduction in recession depth through creeping attachment, with the amount of attachment being linked to the initial recession depth [28]. After a free gingival graft is placed in a specific area, the area may often perceive bridging and/or creeping attachment. Bridging involves some of the grafted tissue remaining healthy over the root, an avascular zone. Creeping attachment occurs as the grafted gingiva migrates coronally in the years post-surgery. These phenomena are commonly observed in cases of narrow recession where the grafts are directly placed over the denuded zone [29]. In a preliminary comparative study by Ito et al., both guided tissue regeneration and free gingival graft procedures showed similar results in terms of root coverage, reduction in gingival recession, and clinical attachment gain. The guided tissue regeneration procedure was noted to offer improved aesthetic outcomes without affecting gingival color or architecture in cases of adjacent facial gingival recession [30]. Sriwil et al. conducted a study comparing the effectiveness of gingival unit graft (GUG) and free gingival graft (FGG) in treating extensive gingival recession and enhancing keratinized tissue. The study results showed that the postoperative root coverage percentage was higher on the GUG side at one and six months, with a significant increase in keratinized tissue one month after surgery. The study suggested that GUG could be a viable approach for augmenting keratinized tissue and addressing recession concerns [31].

## Conclusions

Our review indicates that modifying the FGG procedure could enhance treatment outcomes. The outcomes varied depending on the modified technique employed, encompassing the successful coverage of roots, enhanced matching of gingival color, increased keratinized tissue, promotion of new attachment, and improved healing of the donor site. More studies are recommended, including clinical trials, to assess each modified technique and its outcomes.
